# Attitude toward vital signs monitoring and its predictors among clinical nurses in Saudi Arabia

**DOI:** 10.3389/fpubh.2024.1454851

**Published:** 2024-10-11

**Authors:** Abdualrahman Saeed Alshehry

**Affiliations:** Medical-Surgical Department, College of Nursing, King Saud University, Riyadh, Saudi Arabia

**Keywords:** attitudes, clinical nurses, nursing practice, vital signs, vital signs monitoring, predictor, Saudi Arabia

## Abstract

Positive attitudes toward assessing vital signs are essential in ensuring quality assessments and recognizing patients’ declining conditions. However, few studies have been conducted that examine the attitudes of nurses toward this fundamental nursing skill. This research investigated the predictors of clinical nurses’ attitudes toward vital signs monitoring in identifying the patients’ deteriorating state. A cross-sectional, correlational study was conducted in two hospitals in Saudi Arabia. A sample of 427 clinical nurses was surveyed from February 2023 and April 2023 using a questionnaire on attitudes toward vital signs monitoring. The subscale “key indicators” achieved the highest mean, followed by “workload,” “communication,” and “knowledge.” The hospital where the nurses work (Hospitals 1 and 2), younger age, gender (being male), marital status (being single), clinical area (working in intensive care unit), number of handled patients per shift (handling 11–20 and > 20 patients), and longer years of experience were identified as significant predictors of the nurses’ positive attitude toward vital signs monitoring. This research provides valuable knowledge on which aspect of the attitudes toward vital signs monitoring necessitates educational enhancement among clinical nurses. The factors influencing the clinical nurses’ attitude toward vital signs monitoring reported in this study may be useful for nurse managers and other hospital policymakers in developing focused continuing educational interventions targeting enhanced vital signs monitoring competencies.

## 1 Introduction

The assessment of vital signs (VS) in an appropriate and timely manner is important in ensuring safety of patients (1). Among various healthcare professionals, nurses usually detect patient deterioration through accurate measurement and interpretation of VS (2). Thus, VS assessment is a basic nursing care, and is critical in the detection of patients’ deteriorating conditions (3). The role of nurses in monitoring VS in detecting patient deterioration could effectively prevent adverse events in patient outcomes (4). However, nurses’ poor quality of VS monitoring, recording, and reporting by nurses and the concerns on the precision of current VS monitoring devices pose key challenges to patient safety (4, 5). The substandard quality of VS monitoring, recording, and reporting and the negligence to provide immediate and correct response to patient deterioration are likely associated with increased morbidity, hospital cost, unplanned intensive care, cardiac or pulmonary arrest, and mortality (2, 6). Having positive attitudes toward assessing VS is essential in ensuring quality assessments and in recognizing declining conditions of patients (7). However, few studies had been conducted that examines the attitudes of nurses toward this fundamental nursing skill. Therefore, this study investigated the attitude of clinical nurses on VS monitoring in identifying deteriorating patient condition. The predictors of these attitudes were also examined.

### 1.1 Background

VS are considered critical in predicting patient’s deterioration (8). Patient deterioration is “an evolving, predictable, and symptomatic process of worsening physiology toward critical illness.” (2) VS outside of normal ranges are indicative of clinical deterioration (9). VS assessment, as part of surveillance, determines early warning signals of deterioration and enhanced patient outcomes (6, 8, 10). Performing assessments of VS is an easy, simple and cost-effective method of assessment for patients in any acute care facility (6). VS assessment produces baseline information, monitors changes on patients’ health condition, detects deterioration, and evaluates the effectiveness of various interventions (11). However, the inability of nurses to appropriately and timely recognize and intervene with patients’ deteriorating conditions remains an issue worldwide (12).

Previous investigations have supported the significance of VS monitoring in detecting patients’ deteriorating conditions. Findings from an Australian study revealed that utilizing VS and visual assessment resulted in nurses’ confidence on their abilities to identify patient deterioration, whereas utilizing continuous monitoring equipment showed potential benefits in supporting the detection of deterioration (5). Another study in Australia used qualitative-observational method to examine the VS monitoring practices of nurses and revealed that the five required VS according to the policy were only assessed in 6–21% occasions of VS monitoring (1). Despite the acknowledged significant role of VS in detecting deterioration, earlier studies had reported that some clinical nurses have poor implementation (i.e., monitoring and reporting delays) of these fundamental nursing care. According to the study of Mok et al., nurses seem to monitor VS routinely but often neglect its importance in detecting patients’ deteriorating conditions (4). In addition, a study conducted in UK maternity units reported varying chart formats and warning systems and protocols. In particular, the differences in the normal values of VS based on different systems suggest poor equity in the processes of detecting patient deterioration (13). Another UK study conducted among ward nurses revealed that many inaccurately believe that blood pressure alterations are the initial indicator of deterioration, while nearly half of the surveyed nurses agree that changes in respiratory rate are the least important among the indicators (3). Nurses in New Zealand similarly identified respiratory rate as the most frequently missed VS measured and documented (14). Another review provided an evidence that the most accurate VS that predicts patient deterioration is respiratory rate (15) and compromised respiratory function could lead to ICU admission (16).

The nurses’ poor quality of assessment, untimely monitoring and reporting; the differences in assessment, warning systems, and care protocols; and the need to improve the identification of patient deterioration warrant the necessity to explore nurses’ attitudes toward VS monitoring. Given that VS assessment is considered a basic nursing role in detecting patient deterioration, these factors also call for a need and action for continuous professional development to advance these attitudes. The capacity of nurses to perceive and detect deterioration cues could help categorize clinical signs indicating patient deterioration and assist nurses with effective response. Exploring nurses’ attitudes toward VS monitoring is important for enhancing the knowledge on how they perceive, behave, and respond. It may also result in the formulation of evidence-based interventions and policies upholding the roles of nurses in detecting and reporting deteriorating conditions in the earliest possible time and accurate manner.

## 2 Materials and methods

### 2.1 Design

This quantitative study employed a cross-sectional, correlational design. The cross-sectional design was selected because of its several advantages, particularly its effectiveness in validating or disproving the assumptions delineated in this study. The correlational design utilized in this study involved examining the associations between specific nurses’ demographic characteristics and each subscale of the V-scale. Hence, the utilization of this type of research design enabled the study to examine the distributions of independent or predictor variables, which include the nurses’ demographic characteristics, perceived knowledge and perceived competence, and the dependent or outcome variables characterized by the subscales of the V-scale. This study adhered to the STrengthening the Reporting of OBservational studies in Epidemiology (STROBE) guidelines for cross-sectional studies (Supplementary file 1).

### 2.2 Setting and sampling

The investigation was performed in two hospitals located in Riyadh Province, Saudi Arabia. These hospitals provide emergency, outpatient, and in-patient healthcare services to Saudi and non-Saudi patients. Convenience sampling technique was used to select 427 clinical nurses. We included nurses who were: (1) registered nurses in Saudi Arabia, (2) employed in any of the two hospitals included in the study, and (3) directly involved in patient care. Nurses who do not provide direct care and those who work primarily in the administrative aspect of the hospital were excluded from the study. A *post hoc* power analysis (G*Power) was performed to examine the statistical power achieved by the sample size. The analysis yielded a statistical power of 99.7% at 0.05 significance level and medium effect size, implying that the current sample size had enough power to detect medium differences.

### 2.3 Instrumentation and data collection

Data were obtained using a self-administered questionnaire consisting of two parts: demographic characteristics and a 16-item question on attitudes toward VS monitoring (V-scale), of which written permission was obtained from the copyright holder on October 12, 2022 (3). The demographic section consisted of items tailored to gather information regarding age, gender, marital status, highest educational achievement (diploma in nursing, bachelor’s degree, and master’s degree), clinical area, total clinical experience, estimated average number of patients per shift, and educational lectures/trainings attended on VS. One question on self-reported knowledge on VS assessment (scale of 1 to 10) and one question on perceived competence on VS monitoring (scale of 1 to 10) were also added.

The clinical nurses’ attitudes on VS monitoring for patient deterioration detection were assessed using the V-scale by Mok et al. (3) This tool consists of 16 items designed to measure five components of the nurses’ attitudes, including “key indicators,” “knowledge,” “communication,” “workload,” and “technology.” Under the subscale “key indicators,” the respondents were asked for their agreement with statements such as “SpO2 is a more reliable indicator in reflecting early signs of respiratory dysfunction than respiratory rate.” The items under the subscale “knowledge” included “I can relate VS readings to the physiology and pathophysiology of presenting diseases.” The items under the subscale “communication” included “I am confident to report deteriorating VS in a way that will get a team doctor/ RN in-charge to review the patient.” For the subscale “workload,” the items included “it is time consuming to perform VS monitoring.” The items under the subscale “technology” included “respiratory rate value is usually estimated for stable patients during routine VS monitoring.” Each item in the scale was answered by choosing from 1 “strongly disagree” to 5 “strongly agree.” Scoring was achieved by obtaining the mean score of each dimension. Negatively worded items were reversed coded before calculating the scores. Hence, higher mean implies better attitude. The psychometric properties of the V-scale were reported by Mok et al. (3) The Cronbach’s alpha was 0.71. The exploratory factor analysis revealed a five-factor solution of the V-scale with a total variance of 56.3% (3).

The members of the research team (researcher and research assistant) visited each hospital to coordinate with the nursing director and head of the nursing departments and identify the available time of the nurses. The researchers approached the nurses during their free time. The researchers thoroughly explained the essential study information to the prospective respondents. Adequate time was allotted for questions and answers. The nurses who signified their intention to participate signed the informed consent form and were given the questionnaire. The respondents were instructed to place the filled-out surveys in the boxes placed in designated places in the hospital. Data collection was performed for a 3-month period between February 2023 and April 2023.

### 2.4 Ethical considerations

This research was approved by the Central Institutional Review Board of the Ministry of Health of Saudi Arabia (Approval Number: 2023-0121E). The management of the two hospitals allowed the conduct of the study. Written informed consent was obtained from the respondents before providing them with the questionnaire. The study’s information and the respondents’ right were mentioned during the recruitment phase. The nurses participated voluntarily in the study. Data were treated collectively.

### 2.5 Data analysis

Data were analyzed on SPSS version 22. Descriptive statistics was used to describe the nurses’ demographics and the V-scale answers. Multivariate regression analysis with Wilks’s Lambda was initiated to test the multivariate influence of the nurses’ characteristics to each V-scale’s subscale. Multiple regression analyses were performed on the five V-scale dimensions to assess the demographic predictors of the nurses’ attitude toward VS monitoring. Statistical significance was ascertained below a *p*-value of 0.05.

## 3 Results

### 3.1 Demographic characteristics of participants

Of the 500 surveys distributed, 427 were returned (response rate: 85.4%). Among the 427 surveyed nurses, 53% were employed in Hospital 1 and the remaining were from Hospital 2. The average age was 33.66 (SD = 8.50) years (range = 22–59 years). Most of the nurses were females (88.8%), married (52.7%), BSN graduate (90.5%), and attended training/seminar related to VS in the past 6 months (51.9%). Their average years of experience was 9.56 (SD = 7.37; range = 1–32 years). The highest percentage of respondents was assigned in the surgical department (27.4%), followed by medical department (25.7%), emergency department (19.1%), and intensive care units (14.5%). The lowest belonged to the pediatric department (13.3%). Around 40.7% of the respondents handled an average of 1–10 patients per shift, while 22.0 and 37.3% handled 11–20 and > 20 patients per shift. Regarding self-reported knowledge and competence, the respondents exhibited mean scores of 8.17 (SD = 1.64) and 7.96 (SD = 1.93), respectively.

### 3.2 Results of the descriptive analysis on the V-scale

The clinical nurses’ overall average in the V-scale was 3.47 (SD = 0.53). “Key indicators” achieved the highest mean (mean = 3.76, SD = 0.89), followed by “workload” (mean = 3.64, SD = 0.85), “communication” (mean = 3.58, SD = 1.29), and “knowledge” (mean = 3.41, SD = 0.94). The subscale “technology” was rated as the lowest by the respondents (mean = 3.08, SD = 1.03). In terms of the individual items on the scale, the majority of the respondents were affirmative on the items “respiratory rate value is usually estimated for stable patients during routine VS monitoring” (59.0%), “electronic VS monitoring results in manual monitoring (i.e., counting) of respiratory rate” (55.6%), “I am confident to report deteriorating VS in a way that will get a team RN in-charge or my clinical instructor to review the patient” (64.7%), and “I repeatedly inform the team RN in-charge of VS changes if no prompt actions are acted on” (53.5%). Moreover, the majority of the respondents reported disagreement on the items “VS monitoring is a boring task” (68.5%), “blood pressure is often the first parameter that reflects abnormality when patient condition deteriorates” (68.0%), “respiratory rate is the least important sign of deterioration” (72.6%), and “changes in VS are not interpreted accurately by nurses” (50.2%; Table 1).

**Table 1:** Results of the descriptive analysis on the V-Scale (*n* = 427).

Variable	Strongly disagree/disagree	Neutral	Strongly agree/agree
	*n* (%)	*n* (%)	*n* (%)
Technology (Mean ± SD = 3.08 ± 1.03)			
1. Respiratory rate value is usually estimated for stable patients during routine vital signs monitoring.^a^	81 (33.6)	17 (7.1)	143 (59.3)
2. Electronic vitals monitoring results in casual monitoring (i.e., counting) of respiratory rate.^a^	62 (25.7)	45 (18.7)	134 (55.6)
3. The use of pulse oximetry to monitor SpO2 will reduce the need to count respiratory.^a^	140 (58.1)	33 (13.7)	68 (28.2)
4. I usually record respiratory rate as standard rate between 12 and 20/min if SpO2 is within normal range.^a,b^	119 (49.4)	34 (14.1)	88 (36.5)
Communication (Mean ± SD = 3.58 ± 1.29)			
5. I am confident to report deteriorating vital signs in a way that will get a team RN in-charge or my clinical instructor to review the patient.	53 (22.0)	32 (13.3)	156 (64.7)
6. I will repeatedly inform the team RN in-charge or my clinical instructor of vital sign changes if no prompt actions are acted on.	71 (29.5)	41 (17.0)	129 (53.5)
Workload (Mean ± SD = 3.64 ± 0.85)			
7. It is time consuming to perform vital signs monitoring.^a^	127 (52.7)	56 (23.2)	58 (24.1)
8. Vital signs monitoring is a boring task.^a^	165 (68.5)	49 (20.3)	27 (11.2)
9. Complete and accurate vital signs monitoring is neglected due to time constraints.^a^	102 (42.3)	81 (33.6)	58 (24.1)
10. I feel overwhelmed trying to complete the different frequency of vital signs collection (i.e., hourly, 2 hourly, 4 hourly, etc.) of my patients.^a^	106 (44.0)	56 (23.2)	79 (32.8)
Key indicators (Mean ± SD = 3.76 ± 0.89)			
11. SpO2 is a more reliable indicator in reflecting early signs of respiratory dysfunction than respiratory rate.^a^	117 (48.5)	35 (14.5)	89 (36.9)
12. Blood pressure is often the first parameter that reflects abnormality when a patient deteriorates.^a^	164 (68.0)	39 (16.2)	38 (15.8)
13. Respiratory rate value is the least important sign of deterioration.^a^	175 (72.6)	27 (11.2)	39 (16.2)
Knowledge (Mean ± SD = 3.41 ± 0.94)			
14. I can relate vital signs readings to physiology and pathophysiology of presenting diseases.	54 (22.4)	69 (28.6)	118 (49.0)
15. My knowledge in interpreting vital signs to identify clinical deterioration is limited.^a^	88 (36.5)	85 (35.3)	68 (28.2)
16. Changes in vital signs were not interpreted accurately by nurses (i.e., absence or delay of appropriate nursing actions).^a^	121 (50.2)	80 (33.2)	40 (16.6)

^a^Scores were reversed before computation. ^b^For pediatric nurses, the VS values were changed for pediatric context where the phrase ‘according to the child’s age’ (28) replaced ‘between 12 and 20/min’.

### 3.3 Results of the multivariate regression analysis using Wilks’ lambda test on the subscales of the V-scale

The findings of the multivariate regression on the five subscales of the V-scale revealed that hospital, age, gender, marital status, clinical area, patients per shift, and years of experience had multivariate influences on the subscales (Table 2). The regression model for “technology” [*F* (15, 225) = 5.46, *p* < 0.001], “communication” [*F* (15, 225) = 7.96, *p* < 0.001], “workload” [*F* (15, 225) = 6.42, *p* < 0.001], “key indicators” [*F* (15, 225) = 6.62, *p* < 0.001], and “knowledge” [*F* (15, 225) = 3.31, *p* < 0.001] were statistically significant, accounting for 26.7, 34.7, 30.0, 30.6, and 18.1% of the variance, respectively.

**Table 2:** Results of the multivariate regression analysis using Wilks’ Lambda test on the subscales of the V-scale (*n* = 427).

Predictor variables	Value	*F*	Hypothesis df	Error df	*p*
Hospital	0.72	17.40	5.00	225.00	<0.001***
Age	0.85	8.15	5.00	225.00	<0.001***
Gender	0.95	2.33	5.00	225.00	0.044*
Marital status	0.92	3.93	5.00	225.00	0.002**
Education	0.99	0.49	5.00	225.00	0.787
Clinical area	0.87	6.74	5.00	225.00	<0.001***
Patient/ shift	0.87	6.60	5.00	225.00	<0.001***
Training/ seminars	0.96	1.91	5.00	225.00	0.094
Experience	0.90	4.88	5.00	225.00	<0.001***
Perceived knowledge	0.99	0.49	5.00	225.00	0.785
Perceived competence	0.05	2.01	5.00	225.00	0.078

*Significant at 0.05, **Significant at 0.01, ***Significant at 0.001.

### 3.4 Results of the multiple regression analyses on the subscales of the V-scale

Being employed in Hospital 1 was associated with positive attitudes toward “workload” and “key indicators,” while working in Hospital 2 corresponded to positive attitudes on “technology” and “communication.” Younger age was a predictor of more positive attitudes toward “knowledge” and “technology.” Male gender was associated with positive attitudes toward “technology.” In addition, being single showed positive attitudes toward “communication” and “knowledge.” ICU nurses had significantly better attitudes on the subscales “technology” and “communication” than nurses in the surgical and pediatric departments, respectively. They also had more positive attitudes on “workload” and “key indicator” than the nurses in the medical, surgical, pediatric, and emergency departments. The nurses who attend to 11–20 patients per shift reported poorer attitudes on “technology” and “workload” than those who attend to 1–10 patients. Likewise, the nurses who care for >20 patients per shift had poorer attitudes on “workload” and “knowledge” than those who care for only 1–10 patients per shift. On the contrary, the nurses who provide care for 11–20 and > 20 patients reported better attitudes on “communication” than those who provide care for only 1–10 patients every shift. Longer years of experience was associated with poorer attitudes on “technology” (Table 3).

**Table 3:** Results of the multiple regression analyses on the subscales of the V-scale.

Dependent variable	Predictors	ß	SE	*p*	95% CI	
					Lower	Upper
Technology *R^2^* (Adjusted *R^2^*) = 0.267 (0.218)	Hospital	0.37	0.18	0.038*	0.02	0.72
	Age	0.07	0.01	<0.001***	0.04	0.10
	Gender	−0.48	0.22	0.028*	−0.91	−0.05
	Marital status	−0.21	0.15	0.175	−0.50	0.09
	Education	0.39	0.22	0.073	−0.04	0.83
	Clinical area (Reference: ICU)					
	Medical department	−0.16	0.23	0.491	−0.62	0.30
	Surgical department	−0.61	0.20	0.002**	−1.00	−0.23
	Pediatric department	−0.12	0.25	0.644	−0.60	0.37
	Emergency department	−0.16	0.24	0.515	−0.63	0.32
	Patients/ shift (Reference: 1–10 patients)					
	11–20 patients	−0.49	0.21	0.018*	−0.90	−0.09
	> 20 patients	−0.00	0.22	0.984	−0.43	0.42
	Training/ seminars	0.34	0.14	0.017*	0.06	0.61
	Experience	−0.06	0.02	0.001**	−0.09	−0.02
	Perceived knowledge	0.02	0.08	0.847	−0.15	0.18
	Perceived competence	−0.00	0.08	0.959	−0.15	0.15
Communication *R^2^* (Adjusted *R^2^*) = 0.347 (0.303)	Hospital	1.65	0.21	<0.001***	1.23	2.06
	Age	0.03	0.02	0.115	−0.01	0.06
	Gender	0.22	0.26	0.392	−0.29	0.73
	Marital status	−0.60	0.18	0.001**	−0.95	−0.25
	Education	−0.10	0.26	0.700	−0.61	0.41
	Clinical area (Reference: ICU)					
	Medical department	0.18	0.28	0.503	−0.36	0.73
	Surgical department	−0.17	0.23	0.465	−0.62	0.29
	Pediatric department	−0.71	0.29	0.016*	−1.29	−0.13
	Emergency department	−0.51	0.28	0.075	−1.07	0.05
	Patients/shift (Reference: 1–10 patients)					
	11–20 patients	0.66	0.24	0.008**	0.18	1.14
	> 20 patients	0.70	0.26	0.007**	0.19	1.20
	Training/ seminars	−0.34	0.17	0.042*	−0.66	−0.01
	Experience	−0.03	0.02	0.171	−0.06	0.01
	Perceived knowledge	0.00	0.10	0.991	−0.19	0.19
	Perceived competence	0.13	0.09	0.143	−0.05	0.31
Workload *R^2^* (Adjusted *R^2^*) = 0.300 (0.253)	Hospital	−0.31	0.14	0.033*	−0.59	−0.03
	Age	0.01	0.01	0.447	−0.01	0.03
	Gender	−0.04	0.18	0.803	−0.39	0.30
	Marital status	0.08	0.12	0.521	−0.16	0.32
	Education	0.39	0.18	0.029*	0.04	0.74
	Clinical area (Reference: ICU)					
	Medical department	−0.50	0.19	0.009**	−0.87	−0.12
	Surgical department	−0.64	0.16	<0.001***	−0.95	−0.33
	Pediatric department	−0.51	0.20	0.012*	−0.91	−0.11
	Emergency department	−0.97	0.19	<0.001***	−1.35	−0.58
	Patients/ shift (Reference: 1–10 patients)					
	11–20 patients	−0.84	0.17	<0.001***	−1.17	−0.51
	> 20 patients	−0.39	0.18	0.028*	−0.74	−0.04
	Training/ seminars	0.18	0.11	0.105	−0.04	0.41
	Experience	0.01	0.01	0.671	−0.02	0.03
	Perceived knowledge	0.00	0.07	0.949	−0.13	0.14
	Perceived competence	0.03	0.06	0.660	−0.09	0.15
Key indicators *R^2^* (Adjusted *R^2^*) = 0.306 (0.260)	Hospital	−0.58	0.15	<0.001***	−0.88	−0.29
	Age	−0.01	0.01	0.474	−0.03	0.02
	Gender	−0.05	0.18	0.785	−0.41	0.31
	Marital status	0.10	0.13	0.448	−0.15	0.35
	Education	0.02	0.18	0.903	−0.34	0.39
	Clinical area (Reference: ICU)					
	Medical department	−1.00	0.20	<0.001***	−1.39	−0.62
	Surgical department	−0.67	0.16	<0.001***	−0.99	−0.34
	Pediatric department	−0.54	0.21	0.011*	−0.95	−0.13
	Emergency department	−0.85	0.20	<0.001***	−1.25	−0.45
	Patients/shift (Reference: 1–10 patients)					
	11–20 patients	−0.28	0.17	0.111	−0.62	0.06
	> 20 patients	0.22	0.18	0.234	−0.14	0.58
	Training/ seminars	0.26	0.12	0.026*	0.03	0.49
	Experience	0.01	0.01	0.376	−0.01	0.04
	Perceived knowledge	−0.02	0.07	0.760	−0.16	0.12
	Perceived competence	0.07	0.06	0.260	−0.05	0.20
Knowledge *R^2^* (Adjusted *R^2^*) = 0.181 (0.126)	Hospital	−0.13	0.17	0.463	−0.46	0.21
	Age	0.04	0.01	0.008**	0.01	0.06
	Gender	−0.32	0.21	0.127	−0.73	0.09
	Marital status	−0.30	0.15	0.043*	−0.58	−0.01
	Education	0.14	0.21	0.495	−0.27	0.56
	Clinical area (Reference: ICU)					
	Medical department	0.16	0.22	0.480	−0.28	0.60
	Surgical department	0.16	0.19	0.388	−0.21	0.53
	Pediatric department	0.23	0.24	0.341	−0.24	0.70
	Emergency department	−0.05	0.23	0.831	−0.51	0.41
	Patients/ shift (Reference: 1–10 patients)					
	11–20 patients	−0.35	0.20	0.083	−0.74	0.05
	> 20 patients	−0.48	0.21	0.024*	−0.89	−0.06
	Training/ seminars	0.07	0.13	0.596	−0.19	0.34
	Experience	−0.01	0.02	0.373	−0.04	0.02
	Perceived knowledge	−0.11	0.08	0.157	−0.27	0.04
	Perceived competence	0.18	0.07	0.014*	0.04	0.33

*Significant at 0.05; **Significant at 0.01; ***Significant at 0.001.

## 4 Discussion

This study investigated the clinical nurses’ attitude on VS monitoring patient deterioration detection and its predictors. The findings were significant in at least three aspects. First, the overall scores obtained in the V-scale was modest. Second, average scores were noted with regard to the respondents’ perceived knowledge and competence. Third, the respondents’ demographic characteristics influenced their attitudes.

Among the five subscales of the V-scale, “key indicators” subscale garnered the highest score, while the lowest score was observed in “technology” subscale. The results of an earlier study using the same instrument conducted in Hong Kong varied to some extent (3). The low scores in “technology” reflect the findings of Prgomet and colleagues, who revealed that nurses and physicians indicate their apprehension about using such technology in monitoring patient VS, which affect patient care (5). In the current study, this finding could be supported by the respondents’ agreement on the item “electronic VS monitoring results in manual monitoring,” which may influence nurses’ overall attitude toward “technology” and negatively or positively affect VS monitoring. Even though nurses seem to use technology that results in manual monitoring, they still rely on “key indicators” and apply critical thinking, as evidenced by their disagreement with the two items under the “key indicator” subscale. This finding perhaps influenced their overall average scores in knowledge and competence toward VS monitoring. Furthermore, on the basis of the individual items and in comparison with a prior study, similarities and differences were observed in terms of agreement and disagreement (3), possibly due to the respondents’ demographic characteristics.

The influence of demographics on the nurses’ attitudes toward VS monitoring was statistically significant in this study. For instance, the variables Hospital 1, male, and single were related to positive attitude toward at least one to two subscales of the tool. The variables Hospital 2 and younger age were also related to positive attitude toward specific dimensions of the instrument. Also, marital status of nurses particularly being male was considered significant predictor of having more positive attitude toward VS monitoring. This finding is consistent to the evidence that marriage may have a more protective effect for men compared to their women counterpart including self-rated health (17, 18). However, a recent scoping review with 32 included studies regarding the use of VS to recognize and respond to deteriorating patients has suggested further investigation on the influence of patient and nurse related factors on VS assessment (19). None of the demographic variables influenced the instrument’s five subscales, indicating that the respondents only gave importance on one or two dimensions of the tool, which may be likely influenced by their work setting or areas of practice.

The ICU nurses showed positive attitude across the five subscales of the instrument except for “knowledge.” Interestingly, these nurses did not show much importance to “knowledge” toward VS monitoring, possibly indicating that ICU nurses are knowledgeable and skilled in monitoring VS and thus give less critic to this factor on the instrument. However, a disagreement with the item “changes in VS are not interpreted accurately by nurses” was noted under the “knowledge” subscale. A gap possibly exists in terms of nurses’ knowledge of interpreting VS accurately, as shown by the low score in the “knowledge” subscale. This finding was consistent with that of an earlier study (3).

Another identified variable influencing the respondents’ attitudes was related to the nurse–patient ratio. When the number of patients was equal to or above 11 per shift, poor attitude toward “workload,” “technology,” and “knowledge” was observed. This result was somewhat expected, and other studies attributed this to fatigue, which currently remains an area of concern (9). The nurse–patient ratio is certainly an indicator that influences clinical nurses’ attitude toward VS assessment, as shown in the literature (4). However, another interesting finding was that these nurses demonstrated positive attitude toward “communication,” suggesting that they maintain patient safety by communicating frequently. Lydon et al. found that the respondents’ critical responsibility is to report to their colleagues about patients’ VS and that they feel protected if these are communicated (20).

Another surprising variable that was found significant was that as nurses gain longer years of experience, a poor attitude was noted toward the “technology” dimension of the tool. While experience has been documented to be critical to patient care (21), including VS monitoring, nurses perhaps had a negative experience of using technology. Nurses are possibly frequently exposed to several new or updated technologies that one could not cope up with mastering their use and applying to patient care. However, with the rapid advancement in technology, nurses should be aware of and adapt to the ever-changing healthcare system and technology used in the clinical setting to foster patient safety and care excellence (22, 23). For instance, a qualitative study among intensive care unit (ICU) nurses in the United Kingdom reported that the use of camera-based technology was beneficial as a non-contact approach to VS monitoring of ICU patients (24). Also, findings from a previous qualitative study in the Netherlands, which was participated by 12 surgical ward nurses, revealed that using the technology of wearable devises with continuous VS monitoring was perceived by nurses as helpful and easy to use which may support timely detection of patient deterioration (25). Another qualitative study among nurses reported sustainable use of continuous VS monitoring, three years after its introduction, and perceived that using a wireless device for continuous VS monitoring is the new standard of care at a Dutch general surgical ward (26). Other existing studies recognized the critical role played by healthcare providers, particularly nurses on the early identification and intervention of patients’ deteriorating condition, where a series of actions, such as VS documentation and interpretation, communication, and prompt medical management, may minimize associated adverse events, thereby improving patient outcome (27).

### 4.1 Limitations

This study has some limitations. The cross-sectional design used here did not allow for examination of causal relationships between the variables. In addition, this study was only conducted in two governmental hospitals in Riyadh Province. It also used convenience sampling in sample selection. These factors possibly limited the generalizability of the results. However, post-hoc analysis revealed adequate power of the sample size given the number of tested predictor variables. Therefore, future studies may consider surveying more hospitals, including private and public hospitals, in various geographical locations. The variables “perceived knowledge” and “perceived competence” were only measured using a single 10-point scale question. Future studies may use more established tools to measure these variables.

## 5 Conclusion

This study examined the predictors of clinical nurses’ attitudes on VS monitoring in detecting patient deterioration in two hospitals in Saudi Arabia. The nurses manifested modest level of attitudes toward VS monitoring, with “key indicators” receiving the most positive attitude and “technology” receiving the poorest attitude. The hospital where the nurses work (Hospitals 1 and 2), younger age, gender (being male), marital status (being single), clinical area (working in intensive care unit), number of handled patients per shift (having 11–20, and > 20 patients), and longer years of experience demonstrated multivariate influences on the five dimensions of the nurses’ positive attitude toward VS assessment. This study demonstrated that the nurses’ attitudes toward VS monitoring varied to some degree, as noted by the respondents having average knowledge and competence on such important nursing tasks, with the possible cause being multifaceted. Taken together, these findings highlighted the need for continuing education of nurses, especially in using technology for VS monitoring.

## 6 Implications

Central to patient safety and quality care is the prompt recognition of and intervention to patients’ deteriorating condition. Nurses, who are often the healthcare workers with most contact to the patients, have essential roles in this crucial responsibility. However, the clinical nurses surveyed in this study had only modest attitudes on this important and fundamental nursing responsibility. Their moderate perception of their knowledge and competence in this fundamental skill was also alarming and should be considered in planning and executing interventions to improve and ensure that nurses have positive attitude, adequate knowledge, and excellent skills in VS monitoring. The findings provide valuable knowledge on which aspect of the attitudes toward VS monitoring needs to be improved. The poor attitudes toward technology among nurses send a signal that continuing educational interventions regarding technology in relation to VS monitoring are needed. The poor attitudes toward knowledge related to VS monitoring in identifying clinical deterioration and the moderate level of reported VS monitoring knowledge among nurses also implied the need to improve the knowledge of nurses regarding this important nursing responsibility. Nurse educators may use these information to develop continuous professional development on this fundamental nursing skills. Furthermore, the predictors of the clinical nurses’ attitudes reported in this study may be useful for nurse educators, nurse managers and other hospital policymakers in developing focused interventions targeting enhanced VS monitoring attitudes. For example, older nurses must be encouraged to improve their technological orientation and knowledge on VS monitoring. The nurses in other wards should also be the focus due to their poorer attitudes toward VS monitoring than ICU nurses. Moreover, the nurse–patient ratio must be revisited and considered. An ideal ratio must be observed to avoid its negative effect on the nurses’ attitudes toward VS monitoring.

## Data Availability

The original contributions presented in the study are included in the article/Supplementary material, further inquiries can be directed to the corresponding author.
